# Supplementary material for: The Evolution and Characterization of the RNA Interference Pathways in Lophotrochozoa
Source: Genome Biol Evol. 2024 May 7;16(5):evae098. doi: 10.1093/gbe/evae098 (PMC11114477; doi:10.1093/gbe/evae098)
Supplement: evae098_Supplementary_Data [file evae098_supplementary_data.zip › Supplementary_materials.zip!/selected_miRNAs/NoGe_selected_miRNAs/mirdeep_pdf_files/NMRB01000012.1_Notospermus_geniculatus_strain_Ushimado_scaffold12,_whole_genome_shotgun_sequence_569.pdf]

Provisional ID	: NMRB01000012.1_Notospermus_geniculatus_strain_Ushimado_scaffold12,_whole_genome_shotgun_sequence_569
Score total	: 48425.8
Score for star read(s)	: 3.9
Score for read counts	: 48416.7
Score for mfe	: 2.2
Score for randfold	: 0
Score for cons. seed	: 3
Total read count	: 94979
Mature read count	: 94523
Loop read count	: 0
Star read count	: 456

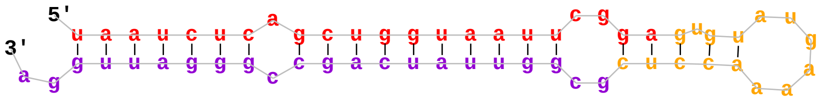

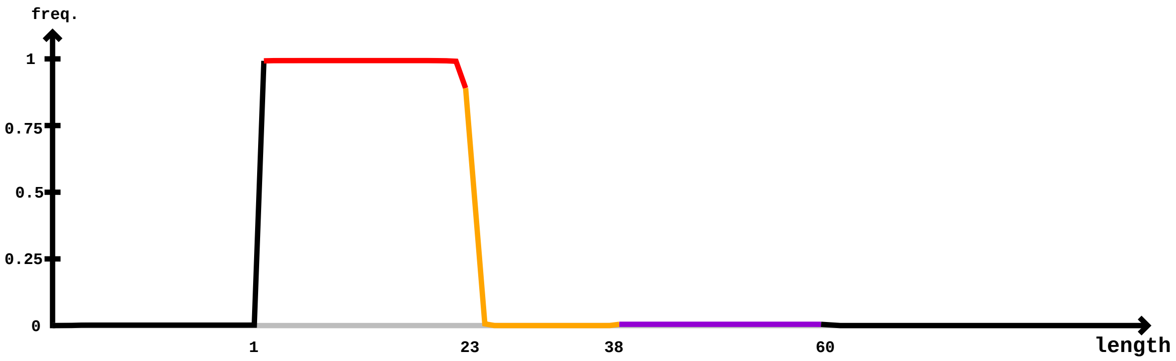


```
                            Mature                          Star
5'- uugccucggccgugucggucuaaucucagcugguaauucggaguguaugaaaaccucgcgguuaucagccgggauuggacggaugcggccaacuaacagcuauucuaaccgu   -3'   obs
    uugccucggccgugucggucuaaucucagcugguaauucggaguguaugaaaaccucgcgguuaucagccgggauuggacggaugcggccaacuaacagcuauucuaaccgu         exp
    .......(((((((((.((((((((((.((((((((((..(((.((......)))))..)))))))))).)))))))))).)))))))))......................   reads   mm   sample
    .ugccucggccgugucgguc............................................................................................   5       0    Mm2
    ..gccucggccgugucgguc............................................................................................   2       0    Mm2
    ...................cuaaucucagcugguaauucgg.......................................................................   1       0    Mm2
    ...................cuaaucucagcugguaauucgga......................................................................   1       0    Mm2
    ....................uaaucucagcugguaauu..........................................................................   2       0    Mm2
    ....................uaaucucagcugguaauuc.........................................................................   4       0    Mm2
    ....................uaaucucagcugguaauucg........................................................................   13      0    Mm2
    ....................uaaucucagcugguaauucgC.......................................................................   5       1    Mm2
    ....................uaaucucagcugguaauucgU.......................................................................   1       1    Mm2
    ....................uaaucuNagcugguaauucgg.......................................................................   1       1    Mm2
    ....................uaaucucagcugguaauucgg.......................................................................   782     0    Mm2
    ....................uaaucucagcugguaaAucgg.......................................................................   5       1    Mm2
    ....................uaaucucGgcugguaauucgg.......................................................................   4       1    Mm2
    ....................uaaucucagcugguaauGcgg.......................................................................   1       1    Mm2
    ....................uaaucucagcugguaauucUg.......................................................................   1       1    Mm2
    ....................uaaucucagcCgguaauucgg.......................................................................   2       1    Mm2
    ....................uaaucucagcugguaauucgA.......................................................................   1       1    Mm2
    ....................uaaucucagcugguaauuUgg.......................................................................   1       1    Mm2
    ....................uaaucCcagcugguaauucgg.......................................................................   1       1    Mm2
    ....................uaaucucagcugguaauCcgg.......................................................................   4       1    Mm2
    ....................uaaucucagcugguaaCucgg.......................................................................   4       1    Mm2
    ....................uaaucAcagcugguaauucgg.......................................................................   1       1    Mm2
    ....................uaaucucagcugguaauAcgg.......................................................................   1       1    Mm2
    ....................uaaucuGagcugguaauucgga......................................................................   1       1    Mm2
    ....................uaaucucagcugguaauCcgga......................................................................   27      1    Mm2
    ....................Naaucucagcugguaauucgga......................................................................   2       1    Mm2
    ....................uUaucucagcugguaauucgga......................................................................   1       1    Mm2
    ....................uaaucNcagcugguaauucgga......................................................................   1       1    Mm2
    ....................uaaucucagcugguaauucUga......................................................................   1       1    Mm2
    ....................uaaucucUgcugguaauucgga......................................................................   2       1    Mm2
    ....................uaaucucagcugguaauucgAa......................................................................   1       1    Mm2
    ....................uaaucucagcuggCaauucgga......................................................................   1       1    Mm2
    ....................uaGucucagcugguaauucgga......................................................................   6       1    Mm2
```

Mature Star

```
uugccucggccgugucggucuaaucucagcugguaauucggaguguaugaaaaccucgcgguuaucagccgggauuggacggaugcggccaacuaacagcuauucuaaccgu
....................uaaucucagcugguaauucggG......................................................................  98    1  Mm2
....................uaaucuAagcugguaauucgga......................................................................  1     1  Mm2
....................uaaucucagcAgguaauucgga......................................................................  6     1  Mm2
....................uaaucucagcugguaaCucgga......................................................................  19    1  Mm2
....................uaaucucagcugguaUuucgga......................................................................  2     1  Mm2
....................uaauAucagcugguaauucgga......................................................................  1     1  Mm2
....................uaaucucagcugguaauuAgga......................................................................  3     1  Mm2
....................uaaucucaCcugguaauucgga......................................................................  1     1  Mm2
....................uaaucucagcugguaauucgCa......................................................................  2     1  Mm2
....................uaaucucagcugguaauucggC......................................................................  16    1  Mm2
....................uaaucucagcugguaauuUgga......................................................................  5     1  Mm2
....................uaaucucagcugguaauucgga......................................................................  4324  0  Mm2
....................uaaucucagcugUuaauucgga......................................................................  1     1  Mm2
....................uaaucucagcugguaaGucgga......................................................................  4     1  Mm2
....................uaaucuNagcugguaauucgga......................................................................  1     1  Mm2
....................uaaucucagcugguaauucCga......................................................................  1     1  Mm2
....................uaaucucagcCgguaauucgga......................................................................  4     1  Mm2
....................uaaucucagcugguaauAcgga......................................................................  17    1  Mm2
....................uaUucucagcugguaauucgga......................................................................  4     1  Mm2
....................uaaucucagcugguaauucAga......................................................................  2     1  Mm2
....................uaaucucaUcugguaauucgga......................................................................  1     1  Mm2
....................uaaucucagcugguaauucggU......................................................................  24    1  Mm2
....................uaaucucagcugguaaAucgga......................................................................  7     1  Mm2
....................uaaucucGgcugguaauucgga......................................................................  16    1  Mm2
....................uaaucucagcugguaauGcgga......................................................................  29    1  Mm2
....................uaaucucagUugguaauucgga......................................................................  1     1  Mm2
....................uGaucucagcugguaauucgga......................................................................  5     1  Mm2
....................uaaucucagcGgguaauucgga......................................................................  7     1  Mm2
....................uaaucucagcugguaauucgUa......................................................................  2     1  Mm2
....................uaaucucagcugguaauuUggag.....................................................................  1     1  Mm2
....................uaGucucagcugguaauucggag.....................................................................  2     1  Mm2
....................uaaucucagcugguaauucggag.....................................................................  25    0  Mm2
....................uaaucucagcugguaauucgUag.....................................................................  1     1  Mm2
....................uaaucucagcugguaauucggaA.....................................................................  37    1  Mm2
....................uaaucucagcugguaauucggaU.....................................................................  457   1  Mm2
....................uaaucucagcugguaauucggaC.....................................................................  21    1  Mm2
....................uaaucucagcugguaauucggaCu....................................................................  11    1  Mm2
....................uaaucucagcugguaauucggagu....................................................................  2     0  Mm2
....................uaauUucagcugguaauucggagu....................................................................  1     1  Mm2
....................uaaucucagcugguaauucggaUu....................................................................  25    1  Mm2
....................uaaucucagcugguaauucggaAu....................................................................  3     1  Mm2
....................uaaucucagcugguaauucggagug...................................................................  2     0  Mm2
.....................aaucucagcugguaauucgg.......................................................................  2     0  Mm2
.....................aaucucagcugguaauucgga......................................................................  5     0  Mm2
........................................................Ugcgguuaucagccgggauugg..................................  1     1  Mm2
........................................................cgcgguuaucagccgggauugg..................................  2     0  Mm2
.........................................................gcgguuaucagccgggauugga.................................  30    0  Mm2
.........................................................gcgguuaucagccgggaCugga.................................  1     1  Mm2
.........................................................gcgguuaucagccgggauuggU.................................  1     1  Mm2
.........................................................gcgguuaucagccgggUuugga.................................  1     1  Mm2
.........................................................gcgguuaucagccgggauuggG.................................  2     1  Mm2
.........................................................gcgguuaucagccgggauuggaU................................  1     1  Mm2

....................uaaucucagcugguaauu..........................................................................  1     0  Mm1
....................uaaucucagcugguaauA..........................................................................  1     1  Mm1
....................uaaucucagcugguaaGu..........................................................................  1     1  Mm1
....................uaaucucagcugguaauuc.........................................................................  1     0  Mm1
....................uaaucucagcugguaauAcgg.......................................................................  2     1  Mm1
....................uaaucucagcugguaauucgg.......................................................................  403   0  Mm1
....................uaaucucagcugguaaAucgg.......................................................................  1     1  Mm1
....................uaaucucUgcugguaauucgg.......................................................................  1     1  Mm1
....................uaaucucagcugguaaGucgg.......................................................................  1     1  Mm1
....................uaaucucagcugguaauGcgg.......................................................................  1     1  Mm1
....................uUaucucagcugguaauucgg.......................................................................  1     1  Mm1
....................uaaucucagcugguaauCcgg.......................................................................  2     1  Mm1
....................uaaucucagcAgguaauucgg.......................................................................  1     1  Mm1
....................uaaucucagcAgguaauucgga......................................................................  4     1  Mm1
....................uaaucucagcugguaaCucgga......................................................................  7     1  Mm1
....................uaaucucagcGgguaauucgga......................................................................  2     1  Mm1
....................uaGucucagcugguaauucgga......................................................................  17    1  Mm1
```

```
                    Mature                Star
uugccucggccgugucggucuaaucucagcugguaauucggaguguaugaaaaccucgcgguuaucagccgggauuggacggaugcggccaacuaacagcuauucuaaccgu
....................uaaucucagcugguaauucggU......................................................................   4      1    Mm1
....................uaaucucagcugguaGuucgga......................................................................   2      1    Mm1
....................uaaucucagcugguaaAucgga......................................................................   1      1    Mm1
....................uaaucucagcCgguaauucgga......................................................................   3      1    Mm1
....................uaaucucGgcugguaauucgga......................................................................   7      1    Mm1
....................uaaucucagcugguaauucUga......................................................................   1      1    Mm1
....................uaaucucagcugguaaGucgga......................................................................   4      1    Mm1
....................uaaucucagcuggAaauucgga......................................................................   1      1    Mm1
....................uaaucucagcugguaauAcgga......................................................................   13     1    Mm1
....................uaaucucagcugguaauCcgga......................................................................   11     1    Mm1
....................Naaucucagcugguaauucgga......................................................................   1      1    Mm1
....................uaaucucagcugguaauucgga......................................................................   1525   0    Mm1
....................uaaucucagcugguaauGcgga......................................................................   8      1    Mm1
....................Gaaucucagcugguaauucgga......................................................................   1      1    Mm1
....................uaaucucagcugguaauucggG......................................................................   8      1    Mm1
....................uaaucucagcugguaauucggaC.....................................................................   3      1    Mm1
....................uaaucucagcugguaauucggag.....................................................................   1      0    Mm1
....................uaaucucagcugguaauucggaA.....................................................................   19     1    Mm1
....................uaaucucagcugguaauucggaU.....................................................................   109    1    Mm1
....................uaaucucagcugguaauucggaUu....................................................................   8      1    Mm1
.....................aaucucagcugguaauucgga......................................................................   1      0    Mm1
.....................Uaucucagcugguaauucgga......................................................................   1      1    Mm1

uugccucggccgugucgguc............................................................................................   7      0    Fm2
...................cuaaucucagcugguaauucgga......................................................................   8      0    Fm2
....................uaaucucagcugguaauu..........................................................................   9      0    Fm2
....................uaaucucagcugguaauAc.........................................................................   1      1    Fm2
....................uaaucucagcugguaauuc.........................................................................   5      0    Fm2
....................uaaucucagcugguaauucg........................................................................   52     0    Fm2
....................uaaucucagcugguaauGcg........................................................................   1      1    Fm2
....................uaaucucagcugguaauAcg........................................................................   1      1    Fm2
....................uaaucucagcugguaaCucgg.......................................................................   2      1    Fm2
....................uaaucucagcugguaauCcgg.......................................................................   7      1    Fm2
....................uaaucucagcugguaauucgC.......................................................................   1      1    Fm2
....................uaaucucagcugguaauAcgg.......................................................................   6      1    Fm2
....................uaaucucagcugguaauucgA.......................................................................   1      1    Fm2
....................uaaucucagcugguaauuUgg.......................................................................   1      1    Fm2
....................uGaucucagcugguaauucgg.......................................................................   1      1    Fm2
....................uaaucucagcugguaauGcgg.......................................................................   6      1    Fm2
....................uaaucucagcugguaaGucgg.......................................................................   6      1    Fm2
....................uaaucucagcugguaauucgg.......................................................................   1189   0    Fm2
....................uaaucucUgcugguaauucgg.......................................................................   1      1    Fm2
....................uaaucucGgcugguaauucgg.......................................................................   28     1    Fm2
....................uaaucucagcugguaauucUg.......................................................................   3      1    Fm2
....................uaaucucagcugguaUuucgg.......................................................................   1      1    Fm2
....................uaaucucagcugguaaAucgg.......................................................................   1      1    Fm2
....................uaaucucagcugguaauuUgga......................................................................   10     1    Fm2
....................uaaucucagcugguCauucgga......................................................................   1      1    Fm2
....................uNaucucagcugguaauucgga......................................................................   2      1    Fm2
....................Caaucucagcugguaauucgga......................................................................   1      1    Fm2
....................uaaucNcagcugguaauucgga......................................................................   1      1    Fm2
....................uaaucCcagcugguaauucgga......................................................................   1      1    Fm2
....................uaaucucagcugAuaauucgga......................................................................   2      1    Fm2
....................uaauUucagcugguaauucgga......................................................................   1      1    Fm2
....................uaauGucagcugguaauucgga......................................................................   1      1    Fm2
....................uaaucuUagcugguaauucgga......................................................................   5      1    Fm2
....................Naaucucagcugguaauucgga......................................................................   9      1    Fm2
....................uaaucucagcuggGaauucgga......................................................................   2      1    Fm2
....................uaaucucagAugguaauucgga......................................................................   1      1    Fm2
....................uaaAcucagcugguaauucgga......................................................................   3      1    Fm2
....................uaaucuGagcugguaauucgga......................................................................   2      1    Fm2
....................uaaucucGgcugguaauucgga......................................................................   53     1    Fm2
....................uaaucucagcugguaauucgga......................................................................   18468  0    Fm2
....................uaaucucagcuAguaauucgga......................................................................   1      1    Fm2
....................uaauAucagcugguaauucgga......................................................................   2      1    Fm2
....................uaaucucagcugguaauucggC......................................................................   8      1    Fm2
....................uaaucucagcugguaauucUga......................................................................   7      1    Fm2
....................uaaucucaUcugguaauucgga......................................................................   3      1    Fm2
....................uaGucucagcugguaauucgga......................................................................   16     1    Fm2
....................uaaucucagcugguGauucgga......................................................................   3      1    Fm2
```

Mature **Star**

```
uugccucggccgugucggucuaaucucagcugguaauucggaguguaugaaaaccucgcgguuaucagccgggauuggacggaugcggccaacuaacagcuauucuaaccgu
....................uaaucucagcugguaauuAgga......................................................................  2     1  Fm2
....................uaaucucagcuggAaauucgga......................................................................  4     1  Fm2
....................uGaucucagcugguaauucgga......................................................................  5     1  Fm2
....................uaaucucagcugguaauGcgga......................................................................  84    1  Fm2
....................uaaucucagcAgguaauucgga......................................................................  41    1  Fm2
....................uaaucucagcugguaauucgCa......................................................................  3     1  Fm2
....................uaNucucagcugguaauucgga......................................................................  1     1  Fm2
....................uaaucucagcugguaaGucgga......................................................................  45    1  Fm2
....................uaaucucagcugguaUuucgga......................................................................  4     1  Fm2
....................uaUucucagcugguaauucgga......................................................................  1     1  Fm2
....................uaaucucUgcugguaauucgga......................................................................  2     1  Fm2
....................uaCucucagcugguaauucgga......................................................................  3     1  Fm2
....................uaaucucagcCgguaauucgga......................................................................  14    1  Fm2
....................uaaucucagcugguaauAcgga......................................................................  84    1  Fm2
....................uaaucucagcugguaauucAga......................................................................  2     1  Fm2
....................uaaucucagcugguaaCucgga......................................................................  58    1  Fm2
....................Aaaucucagcugguaauucgga......................................................................  1     1  Fm2
....................uaaucucagcugCuaauucgga......................................................................  1     1  Fm2
....................uCaucucagcugguaauucgga......................................................................  1     1  Fm2
....................uaaucucagcuggCaauucgga......................................................................  3     1  Fm2
....................uaaucAcagcugguaauucgga......................................................................  1     1  Fm2
....................uaaCcucagcugguaauucgga......................................................................  2     1  Fm2
....................uaaucuAagcugguaauucgga......................................................................  4     1  Fm2
....................uaaucucagUugguaauucgga......................................................................  1     1  Fm2
....................uaaucucagcuUguaauucgga......................................................................  5     1  Fm2
....................uaaucucagcugguaauucgUa......................................................................  8     1  Fm2
....................uaaucucagcugguaaAucgga......................................................................  31    1  Fm2
....................uaaucucagcugguaauCcgga......................................................................  102   1  Fm2
....................uaaucucagcugguaauucggG......................................................................  562   1  Fm2
....................uaaucucagcugguaauucggU......................................................................  24    1  Fm2
....................uUaucucagcugguaauucgga......................................................................  3     1  Fm2
....................uaaucucagcGgguaauucgga......................................................................  28    1  Fm2
....................uaaucucagcugguUauucgga......................................................................  3     1  Fm2
....................uaaucucagcugguaauGcggag.....................................................................  2     1  Fm2
....................uaaucucagcGgguaauucggag.....................................................................  1     1  Fm2
....................uaaucucagcugguaauCcggag.....................................................................  1     1  Fm2
....................uaaucucagcugguaaGucggag.....................................................................  1     1  Fm2
....................uaaucucagcugguaauucgAag.....................................................................  1     1  Fm2
....................uaaucucagcugguaauucggag.....................................................................  93    0  Fm2
....................uaaucucagcugguaauucggaA.....................................................................  38    1  Fm2
....................uaaucucUgcugguaauucggag.....................................................................  1     1  Fm2
....................uaaucucagcAgguaauucggag.....................................................................  1     1  Fm2
....................uaaucucagcugguaauucggaC.....................................................................  49    1  Fm2
....................uaaucucagcugguaauucggaU.....................................................................  1516  1  Fm2
....................uaaucucagcugguaauucggaUu....................................................................  196   1  Fm2
.....................aaucucagcugguaauucgga......................................................................  17    0  Fm2
.....................aaucucagcugguaaGucgga......................................................................  1     1  Fm2
.....................aaucucagcugguaauucggG......................................................................  1     1  Fm2
.....................aaucucagcugguaauucggaU.....................................................................  2     1  Fm2
......................aucucagcugguaauucgga......................................................................  2     0  Fm2
.........................................................gcgguuaucagccgggUuugga.................................  1     1  Fm2
.........................................................gcgguuaucagccgggauugga.................................  35    0  Fm2
.........................................................gcgguuaucagccgggaAugga.................................  1     1  Fm2
.........................................................gcgguuaucagccgggauuggG.................................  2     1  Fm2
.........................................................gcgguuaucagccgggauuggaU................................  10    1  Fm2

uugccucggccgugucgguc............................................................................................  8     0  Fm1
..gccucggccgugucgguc............................................................................................  2     0  Fm1
....................uaaucucagcugguaauu..........................................................................  3     0  Fm1
....................uaaucucagcugguaauucg........................................................................  14    0  Fm1
....................uaaucucGgcugguaauucgg.......................................................................  5     1  Fm1
....................uaaucucagcugguaauCcgg.......................................................................  9     1  Fm1
....................uaaucucagcCgguaauucgg.......................................................................  2     1  Fm1
....................uaaucucagUugguaauucgg.......................................................................  1     1  Fm1
....................uaaucCcagcugguaauucgg.......................................................................  1     1  Fm1
....................uaaucucaUcugguaauucgg.......................................................................  1     1  Fm1
....................uaaucucagcAgguaauucgg.......................................................................  3     1  Fm1
....................uaaucucagcugguaauucgC.......................................................................  5     1  Fm1
....................uaaucucagcugguaauucgg.......................................................................  1423  0  Fm1
....................uaaucucagcugguaauAcgg.......................................................................  5     1  Fm1
```

Mature Star

```
uugccucggccgugucggucuaaucucagcugguaauucggaguguaugaaaaccucgcgguuaucagccgggauuggacggaugcggccaacuaacagcuauucuaaccgu
....................uaaucucagcugguaauGcgg.......................................................................	7	1	Fm1
....................Caaucucagcugguaauucgg.......................................................................	1	1	Fm1
....................uaaucucagcGgguaauucgg.......................................................................	2	1	Fm1
....................Naaucucagcugguaauucgg.......................................................................	1	1	Fm1
....................uaaucucagcugguaauucgA.......................................................................	1	1	Fm1
....................uaaucucagcugguaauucgU.......................................................................	1	1	Fm1
....................uaaucucagcugguaauuAgg.......................................................................	1	1	Fm1
....................uaaucucagcugguaauuUgg.......................................................................	2	1	Fm1
....................uaaucucagcugguaUuucgg.......................................................................	1	1	Fm1
....................uaaucucagcugguaaCucgg.......................................................................	8	1	Fm1
....................uaaucucagcugguaaAucgg.......................................................................	2	1	Fm1
....................uaaucucagcAgguaauucgga......................................................................	10	1	Fm1
....................uaaAcucagcugguaauucgga......................................................................	2	1	Fm1
....................uGaucucagcugguaauucgga......................................................................	3	1	Fm1
....................uaaucucagcCgguaauucgga......................................................................	6	1	Fm1
....................uaaucucagcugguaauucgUa......................................................................	1	1	Fm1
....................uaaucucagcugguaauucgga......................................................................	5864	0	Fm1
....................uaaucucagcugguaaAucgga......................................................................	13	1	Fm1
....................uaaucucagcugguaaCucgga......................................................................	17	1	Fm1
....................uaaucucagcugguaauucAga......................................................................	2	1	Fm1
....................uaaucucGgcugguaauucgga......................................................................	52	1	Fm1
....................uaaucucagcugguaauAcgga......................................................................	27	1	Fm1
....................uaaucucagGugguaauucgga......................................................................	1	1	Fm1
....................uaaucucagcugguaauucggU......................................................................	34	1	Fm1
....................uaaucucagcugguaauuUgga......................................................................	4	1	Fm1
....................uaaucucagcugguaauucggG......................................................................	173	1	Fm1
....................uaaucucagcugAuaauucgga......................................................................	1	1	Fm1
....................uaaucucagcugguaUuucgga......................................................................	1	1	Fm1
....................uaaucucagcugguaauucggC......................................................................	25	1	Fm1
....................uaGucucagcugguaauucgga......................................................................	7	1	Fm1
....................uaaucucagcuggCaauucgga......................................................................	2	1	Fm1
....................uaaucucagcugguaauGcgga......................................................................	24	1	Fm1
....................Aaaucucagcugguaauucgga......................................................................	1	1	Fm1
....................uaaucucagcGgguaauucgga......................................................................	7	1	Fm1
....................uaaucucagcuggAaauucgga......................................................................	2	1	Fm1
....................uaUucucagcugguaauucgga......................................................................	1	1	Fm1
....................uaaucuUagcugguaauucgga......................................................................	1	1	Fm1
....................uaaucucagUugguaauucgga......................................................................	2	1	Fm1
....................uaaucucUgcugguaauucgga......................................................................	1	1	Fm1
....................uaaucucagcugguaauucUga......................................................................	1	1	Fm1
....................Caaucucagcugguaauucgga......................................................................	1	1	Fm1
....................uaaucucagcugguaauCcgga......................................................................	32	1	Fm1
....................uaaucucaUcugguaauucgga......................................................................	6	1	Fm1
....................uaaucucagcugguaaGucgga......................................................................	17	1	Fm1
....................uaaucNcagcugguaauucgga......................................................................	2	1	Fm1
....................uaaucucagcugguaauucggaA.....................................................................	55	1	Fm1
....................uaaucucGgcugguaauucggag.....................................................................	5	1	Fm1
....................uaaucucagcugguaauucggaU.....................................................................	650	1	Fm1
....................uaaucucagcugguaauucggaC.....................................................................	95	1	Fm1
....................uaaucucagcugguaauucggag.....................................................................	36	0	Fm1
....................uaaucucagcugguaauucggaUu....................................................................	110	1	Fm1
....................uaaucucagcugguaauucggaCu....................................................................	27	1	Fm1
....................uaaucucagcugguaauucggaAu....................................................................	3	1	Fm1
.....................aaucucagcugCuaauucgg.......................................................................	1	1	Fm1
.....................aaucucagcugguaauucgg.......................................................................	1	0	Fm1
.....................aaucucagcugguaauucgga......................................................................	3	0	Fm1
.......................ucucagcugguaauucgga......................................................................	1	0	Fm1
..........................................guguaugaaaaccucgcgguuaucagccgAgauugga.................................	1	1	Fm1
..........................................guguaugaaaaccucgcgguuaucagccgggauugga.................................	1	0	Fm1
.........................................................gcgguuaucagccgggauugg..................................	3	0	Fm1
.........................................................gcgguuaucagccgggauuggU.................................	2	1	Fm1
.........................................................gcgguuaucagccgggaCugga.................................	1	1	Fm1
.........................................................gcgguuaucagccgUgauugga.................................	1	1	Fm1
.........................................................gcgguuaucagccgggaAugga.................................	3	1	Fm1
.........................................................gcgguuaucagccgggaGugga.................................	2	1	Fm1
.........................................................gcgguuaucagccgggauugga.................................	280	0	Fm1
.........................................................gcgguuaucagccgggauuggC................................	1	1	Fm1
.........................................................gcgguuaucaCccgggauugga.................................	1	1	Fm1
.........................................................gcgguuauGagccgggauugga.................................	1	1	Fm1
.........................................................gcgguuaucaAccgggauugga.................................	1	1	Fm1
```

Mature **Star**

```
uugccucggccgugucggucuaaucucagcugguaauucggaguguaugaaaaccucgcgguuaucagccgggauuggacggaugcggccaacuaacagcuauucuaaccgu
.........................................................gcgguuaucaUccgggauugga.................................    1    1    Fm1
.........................................................gcgguuaucagccgggauuggG.................................    11   1    Fm1
.........................................................gcgguuaucagccggAauugga.................................    1    1    Fm1
.........................................................gcgguuaucagccgggauuggaU................................    3    1    Fm1
..........................................................cgguuaucagccgggauugga.................................    2    0    Fm1

uugccucggccAugucgguc............................................................................................    1    1    Mm3
uugccucggccgugucgguc............................................................................................    1    0    Mm3
.ugccucggccAugucgguc............................................................................................    5    1    Mm3
.ugccucggccgugucgguc............................................................................................    1    0    Mm3
..gccucggccgugucgguc............................................................................................    2    0    Mm3
...................cuaaucucagcugguaauucgga......................................................................    4    0    Mm3
....................uaaucucagcugguaauu..........................................................................    2    0    Mm3
....................uaaucucagcugguaauuc.........................................................................    11   0    Mm3
....................uaaucucagcugguaauucg........................................................................    24   0    Mm3
....................uaaucucGgcugguaauucg........................................................................    1    1    Mm3
....................uaGucucagcugguaauucgg.......................................................................    1    1    Mm3
....................uaaucucagcugguaauucgA.......................................................................    2    1    Mm3
....................uaaucucagcugguaauucgC.......................................................................    4    1    Mm3
....................uaaucucagcugguaauucgU.......................................................................    2    1    Mm3
....................uaaucucagcAgguaauucgg.......................................................................    3    1    Mm3
....................uaaucucagcuggAaauucgg.......................................................................    1    1    Mm3
....................uGaucucagcugguaauucgg.......................................................................    2    1    Mm3
....................uaaucucagcugguaauCcgg.......................................................................    4    1    Mm3
....................uaaucucagcugguaauucgg.......................................................................    897  0    Mm3
....................uaaucuUagcugguaauucgg.......................................................................    1    1    Mm3
....................uaaucucagcugguaaGucgg.......................................................................    4    1    Mm3
....................uaaucucagcugguaauuGgg.......................................................................    1    1    Mm3
....................uaaucucagcugguaaCucgg.......................................................................    6    1    Mm3
....................Naaucucagcugguaauucgg.......................................................................    1    1    Mm3
....................uaaucucagcugguaauAcgg.......................................................................    6    1    Mm3
....................uaaucucagcugguaauGcgg.......................................................................    8    1    Mm3
....................uaaucucagcGgguaauucgg.......................................................................    1    1    Mm3
....................uaaucucagcuCguaauucgga......................................................................    1    1    Mm3
....................uaCucucagcugguaauucgga......................................................................    1    1    Mm3
....................uaaucucGgcugguaauucgga......................................................................    19   1    Mm3
....................uaauUucagcugguaauucgga......................................................................    1    1    Mm3
....................uaaucucagcugguaauucAga......................................................................    1    1    Mm3
....................uaaucucagUugguaauucgga......................................................................    1    1    Mm3
....................uaaucucagcugguaauucUga......................................................................    1    1    Mm3
....................uaaAcucagcugguaauucgga......................................................................    2    1    Mm3
....................uaaucucagcugguaaAucgga......................................................................    10   1    Mm3
....................uaaucucagcugguaauucgAa......................................................................    4    1    Mm3
....................uaaucCcagcugguaauucgga......................................................................    4    1    Mm3
....................uaaucucagcuggCaauucgga......................................................................    1    1    Mm3
....................uaaucucagcugguaauCcgga......................................................................    32   1    Mm3
....................uaaucucagcugguaaCucgga......................................................................    21   1    Mm3
....................uaaucNcagcugguaauucgga......................................................................    1    1    Mm3
....................uaGucucagcugguaauucgga......................................................................    8    1    Mm3
....................uaaucucagcAgguaauucgga......................................................................    11   1    Mm3
....................uaaucucagcugguaauucggC......................................................................    13   1    Mm3
....................uaaucucagcGgguaauucgga......................................................................    8    1    Mm3
....................uaaucucagcugguaauGcgga......................................................................    28   1    Mm3
....................uaaucucagcugguaauucggU......................................................................    30   1    Mm3
....................uaaucucagcugguaauuUgga......................................................................    2    1    Mm3
....................uaaucucagcugguaauucgCa......................................................................    5    1    Mm3
....................uaaCcucagcugguaauucgga......................................................................    3    1    Mm3
....................uCaucucagcugguaauucgga......................................................................    1    1    Mm3
....................uaaucucagcuggGaauucgga......................................................................    2    1    Mm3
....................uaaucucagcugguaauucgUa......................................................................    2    1    Mm3
....................uaaucucagcCgguaauucgga......................................................................    2    1    Mm3
....................uaaucucagcugguaaGucgga......................................................................    12   1    Mm3
....................uaaucucagcugguGauucgga......................................................................    1    1    Mm3
....................uaaucucagcugguaauucggG......................................................................    92   1    Mm3
....................uUaucucagcugguaauucgga......................................................................    1    1    Mm3
....................Aaaucucagcugguaauucgga......................................................................    2    1    Mm3
....................uaaucucaUcugguaauucgga......................................................................    3    1    Mm3
....................uaaucucagcuggAaauucgga......................................................................    3    1    Mm3
....................uaaucuAagcugguaauucgga......................................................................    1    1    Mm3
....................uaaucAcagcugguaauucgga......................................................................    2    1    Mm3
```

Mature Star

```
uugccucggccgugucggucuaaucucagcugguaauucggaguguaugaaaaccucgcgguuaucagccgggauuggacggaugcggccaacuaacagcuauucuaaccgu
....................Naaucucagcugguaauucgga......................................................................   2      1   Mm3
....................uaaucucagcugguaauAcgga......................................................................   22     1   Mm3
....................uaaucucagcugguaauucgga......................................................................   5838   0   Mm3
....................uaaucucagcugguaauucggaU.....................................................................   388    1   Mm3
....................uaaucucagcugguaauucggaC.....................................................................   14     1   Mm3
....................uaaucucagcugguaauucggaA.....................................................................   35     1   Mm3
....................uaaucucagcugguaauucggag.....................................................................   16     0   Mm3
....................uaaucucagcugguaauucggaAu....................................................................   5      1   Mm3
....................uaaucucagcugguaauucggagu....................................................................   3      0   Mm3
....................uaaucucagcugguaauucggaCu....................................................................   10     1   Mm3
....................uaaucucagcugguaauucggaUu....................................................................   39     1   Mm3
....................uaaucucagcugguaauucggagugu..................................................................   1      0   Mm3
.....................aaucucagcugguaauucgga......................................................................   5      0   Mm3
.....................aaucucagcugguaauucggaU.....................................................................   3      1   Mm3
.........................................................gcgguuaucagccgggauugg..................................   1      0   Mm3
.........................................................gcgguuaucagccgggauugga.................................   9      0   Mm3
.........................................................gcgguuaucagccgggauuggaU................................   1      1   Mm3

uugccucggccgugucggu.............................................................................................   3      0   Fm3
uugccucggccgugucgguc............................................................................................   26     0   Fm3
uugccucggccguguCguc............................................................................................   1      1   Fm3
.ugccucggccgugucggGc............................................................................................   1      1   Fm3
.ugccucggccgugucgguc............................................................................................   79     0   Fm3
.ugccuUggccgugucgguc............................................................................................   1      1   Fm3
.ugccucggccAugucgguc............................................................................................   1      1   Fm3
.ugccucggccguguUgguc............................................................................................   1      1   Fm3
..gccucggccgugucgguc............................................................................................   4      0   Fm3
...................cuaaucucagcugguaauucgga......................................................................   3      0   Fm3
....................uaaucucagcugguaauu..........................................................................   13     0   Fm3
....................uaaucucagcugguaauuc.........................................................................   22     0   Fm3
....................uaaucucGgcugguaauucg........................................................................   1      1   Fm3
....................uaaucucagcugguaauucg........................................................................   53     0   Fm3
....................uaaucucagcugguaaCucg........................................................................   1      1   Fm3
....................uaaucucagUugguaauucg........................................................................   1      1   Fm3
....................uaaucucagcuUguaauucgg.......................................................................   2      1   Fm3
....................uaaucucagcugguaauucgg.......................................................................   4512   0   Fm3
....................uaaucucaUcugguaauucgg.......................................................................   4      1   Fm3
....................uaaucucagcuggAaauucgg.......................................................................   1      1   Fm3
....................uaaCcucagcugguaauucgg.......................................................................   1      1   Fm3
....................uaaucucagcugguaauucgU.......................................................................   1      1   Fm3
....................uaaucucagcugguaaGucgg.......................................................................   14     1   Fm3
....................uaaucucagcugguaauGcgg.......................................................................   34     1   Fm3
....................Gaaucucagcugguaauucgg.......................................................................   1      1   Fm3
....................uaauUucagcugguaauucgg.......................................................................   1      1   Fm3
....................uaaucucagcuggGaauucgg.......................................................................   1      1   Fm3
....................uaaucucagcCgguaauucgg.......................................................................   3      1   Fm3
....................uaaucucagcugguaGuucgg.......................................................................   2      1   Fm3
....................uaaucuUagcugguaauucgg.......................................................................   3      1   Fm3
....................Caaucucagcugguaauucgg.......................................................................   1      1   Fm3
....................uaaucucagcGgguaauucgg.......................................................................   8      1   Fm3
....................uaaucucagcugguaauCcgg.......................................................................   22     1   Fm3
....................uaaucucagcugguaaCucgg.......................................................................   13     1   Fm3
....................uaaucucagcugguaauAcgg.......................................................................   30     1   Fm3
....................uaaucucagcugguaaAucgg.......................................................................   6      1   Fm3
....................uaGucucagcugguaauucgg.......................................................................   3      1   Fm3
....................uaaucucUgcugguaauucgg.......................................................................   2      1   Fm3
....................uaaucucagcugguaauucCg.......................................................................   1      1   Fm3
....................uaaucucagcuCguaauucgg.......................................................................   1      1   Fm3
....................uaaucCcagcugguaauucgg.......................................................................   2      1   Fm3
....................uaaucucagcugguaauucAg.......................................................................   2      1   Fm3
....................uGaucucagcugguaauucgg.......................................................................   4      1   Fm3
....................uaaucucagcugguaauucgA.......................................................................   2      1   Fm3
....................uaaucucGgcugguaauucgg.......................................................................   3      1   Fm3
....................uaaucucagcAgguaauucgg.......................................................................   3      1   Fm3
....................uaaucucagcugguaauuGgga......................................................................   3      1   Fm3
....................uaaucuNagcugguaauucgga......................................................................   1      1   Fm3
....................uaaucucagcugguaauAcgga......................................................................   233    1   Fm3
....................uaaucucagcugguUauucgga......................................................................   1      1   Fm3
....................uaaCcucagcugguaauucgga......................................................................   9      1   Fm3
....................uaaucCcagcugguaauucgga......................................................................   7      1   Fm3
```

```
                    Mature                                   Star
uugccucggccgugucggucuaaucucagcugguaauucggaguguaugaaaaccucgcgguuaucagccgggauuggacggaugcggccaacuaacagcuauucuaaccgu
....................uaaucucagcugguaauCcgga......................................................................  247    1  Fm3
....................uaaucNcagcugguaauucgga......................................................................  6      1  Fm3
....................uaaucucagcugguaauucgga......................................................................  38220  0  Fm3
....................uaaucucagcugguaCuucgga......................................................................  4      1  Fm3
....................uaaucAcagcugguaauucgga......................................................................  2      1  Fm3
....................uaaucucagcCgguaauucgga......................................................................  33     1  Fm3
....................uaaucucagcuggCaauucgga......................................................................  5      1  Fm3
....................uaaucucagcugguaauucgAa......................................................................  2      1  Fm3
....................uaaucucagcugguGauucgga......................................................................  14     1  Fm3
....................uaaucucaUcugguaauucgga......................................................................  10     1  Fm3
....................uaaucucagcugguaauucggC......................................................................  2      1  Fm3
....................uaaucucagcugguaUuucgga......................................................................  12     1  Fm3
....................uaaucucagcugguaauGcgga......................................................................  171    1  Fm3
....................Naaucucagcugguaauucgga......................................................................  13     1  Fm3
....................uaaucucagcugguaauuAgga......................................................................  5      1  Fm3
....................Caaucucagcugguaauucgga......................................................................  6      1  Fm3
....................uaaucucagcugguaaAucgga......................................................................  81     1  Fm3
....................uaaucGcagcugguaauucgga......................................................................  2      1  Fm3
....................uaaucucagcugAuaauucgga......................................................................  1      1  Fm3
....................uaaucucagcuCguaauucgga......................................................................  5      1  Fm3
....................uaaucuGagcugguaauucgga......................................................................  4      1  Fm3
....................uaauUucagcugguaauucgga......................................................................  5      1  Fm3
....................uaaucucagcugUuaauucgga......................................................................  9      1  Fm3
....................uaaucucagAugguaauucgga......................................................................  6      1  Fm3
....................uaaucucagcugguCauucgga......................................................................  3      1  Fm3
....................uaaucucagcugguaauucCga......................................................................  5      1  Fm3
....................uaaucuUagcugguaauucgga......................................................................  6      1  Fm3
....................uGaucucagcugguaauucgga......................................................................  16     1  Fm3
....................uaaucucagcugguaauucgCa......................................................................  4      1  Fm3
....................uaaucucaAcugguaauucgga......................................................................  3      1  Fm3
....................uaaucucagUugguaauucgga......................................................................  7      1  Fm3
....................uaaucucGgcugguaauucgga......................................................................  65     1  Fm3
....................uaaucucagcugguaauucUga......................................................................  17     1  Fm3
....................uUaucucagcugguaauucgga......................................................................  4      1  Fm3
....................uaaucucagcuggAaauucgga......................................................................  16     1  Fm3
....................uaaucucagcugguaauucgUa......................................................................  8      1  Fm3
....................uaaucucagcAgguaauucgga......................................................................  61     1  Fm3
....................uaaucucagcugguaauuUgga......................................................................  21     1  Fm3
....................uaaAcucagcugguaauucgga......................................................................  2      1  Fm3
....................uaaucucagcGgguaauucgga......................................................................  43     1  Fm3
....................uCaucucagcugguaauucgga......................................................................  1      1  Fm3
....................Aaaucucagcugguaauucgga......................................................................  4      1  Fm3
....................uaaucucagcuggGaauucgga......................................................................  4      1  Fm3
....................uaaucucagcugguaaCucgga......................................................................  127    1  Fm3
....................uaaucucCgcugguaauucgga......................................................................  3      1  Fm3
....................Gaaucucagcugguaauucgga......................................................................  1      1  Fm3
....................uaaucucUgcugguaauucgga......................................................................  11     1  Fm3
....................uaaucucagcugCuaauucgga......................................................................  2      1  Fm3
....................uaaucucagcugguaauucggG......................................................................  5      1  Fm3
....................uaaucucagcugguaauucggU......................................................................  34     1  Fm3
....................uaauGucagcugguaauucgga......................................................................  7      1  Fm3
....................uaaucucagcuAguaauucgga......................................................................  5      1  Fm3
....................uaaucucagcugguaauucAga......................................................................  8      1  Fm3
....................uaaucuAagcugguaauucgga......................................................................  4      1  Fm3
....................uaGucucagcugguaauucgga......................................................................  28     1  Fm3
....................uaauAucagcugguaauucgga......................................................................  8      1  Fm3
....................uaCucucagcugguaauucgga......................................................................  3      1  Fm3
....................uaaucucagcugguaGuucgga......................................................................  2      1  Fm3
....................uaaucucagcugguaaGucgga......................................................................  103    1  Fm3
....................uaaucucagcuUguaauucgga......................................................................  14     1  Fm3
....................uaaCcucagcugguaauucggag.....................................................................  1      1  Fm3
....................uaaucucagcugguaauGcggag.....................................................................  1      1  Fm3
....................uaaucucagcugguaauucggaA.....................................................................  105    1  Fm3
....................uaaucucagcugguaauucggag.....................................................................  86     0  Fm3
....................uaaucucagcugguaauucggaU.....................................................................  1749   1  Fm3
....................uaaucucagcugguaauucggaC.....................................................................  48     1  Fm3
....................uaaucucagcugguaauucggaAu....................................................................  10     1  Fm3
....................uaaucucagcugguaauucggaUu....................................................................  130    1  Fm3
....................uaGucucagcugguaauucggagu....................................................................  1      1  Fm3
....................uaaucucagcugguaauucggaCu....................................................................  14     1  Fm3
```

```
                    Mature                                Star
uugccucggccgugucggucuaaucucagcugguaauucggaguguaugaaaaccucgcgguuaucagccgggauuggacggaugcggccaacuaacagcuauucuaaccgu
.....................Uaucucagcugguaauucgga......................................................................    5    1    Fm3
.....................aaucucagcugguaCuucgga......................................................................    1    1    Fm3
.....................aaucucagcugguaauucgga......................................................................    24   0    Fm3
.....................aaucucagcugguaauucggag.....................................................................    1    0    Fm3
.....................aaucucagcugguaauucggaU.....................................................................    4    1    Fm3
.....................aaucucagcugguaauucggaA.....................................................................    1    1    Fm3
......................aucucaCcugguaauucgga......................................................................    1    1    Fm3
......................aucucagcugguaauucggaUu....................................................................    4    1    Fm3
........................cucagcugguaauucUga......................................................................    7    1    Fm3
........................cucagcugguaauucgga......................................................................    4    0    Fm3
.........................................................gcgguuaucagccgggaGugga.................................    1    1    Fm3
.........................................................gcgguuaucagAcgggauugga.................................    1    1    Fm3
.........................................................gcgguuaucagccgggCuugga.................................    1    1    Fm3
.........................................................gcgguuaucagccgggauugga.................................    40   0    Fm3
```
